# Correction: Hansen et al. Two-Year Results of 0.01% Atropine Eye Drops and 0.1% Loading Dose for Myopia Progression Reduction in Danish Children: A Placebo-Controlled, Randomized Clinical Trial. *J. Pers. Med.* 2024, *14*, 175

**DOI:** 10.3390/jpm15120628

**Published:** 2025-12-17

**Authors:** Niklas Cyril Hansen, Anders Hvid-Hansen, Flemming Møller, Toke Bek, Dorte Ancher Larsen, Nina Jacobsen, Line Kessel

**Affiliations:** 1 Department of Ophthalmology, Copenhagen University Hospital—Rigshospitalet-Glostrup, DK-2600 Glostrup, Denmark; 2 Department of Ophthalmology, University Hospital of Southern Denmark—Vejle Hospital, DK-7100 Vejle, Denmark; 3 Department of Ophthalmology, Aarhus University Hospital, DK-8200 Aarhus N, Denmark; 4 Department of Clinical Medicine, University of Copenhagen, DK-2200 København N, Denmark

## Error in Figure 1

In the original publication [1], there was a mistake in Figure 1 as published. The Consolidated Standards of Reporting (CONSORT) flow-diagram erroneously stated that “29, 32, and 33 participants completed the 18-month visit in the placebo, 0.01%, and 0.1% loading dose groups, respectively”. This should instead have read, “at the 18-month visit, 29, 32, and 32 participants were in the placebo, 0.01%, and 0.1% loading dose groups, respectively”. Likewise, Figure 1 states that “28, 32, and 32 participants completed the 24-month visit in the placebo, 0.01%, and 0.1% loading dose group, respectively”. This should instead have read “29, 31, and 32 participants who completed the 24-month visit”. The corrected Figure 1 appears below.

## Text Correction

The second to last sentence of the first paragraph in Section 3 has been corrected as follows:

“Ultimately, 92 (95%) participants completed the two-year visit, of which 32 (35%) were in the 0.1% loading dose group, 31 (34%) were in the 0.01% group, and 29 (31%) were in the placebo group (Figure 1).”

## Error in Table 3

In Table 3, regarding the 24-month visit, the number of participants (total N) in the placebo and 0.01% groups has been corrected to 29 and 31, respectively, as illustrated in this updated version of Table 3:

The authors state that the scientific conclusions are unaffected. The errors are due to mischaracterization during the writing and illustration process of the paper; neither the effect estimates of the examined ophthalmic parameters or the conclusions of this study are in any way affected. This correction was approved by the Academic Editor. The original publication has also been updated.

## Figures and Tables

**Figure 1:**
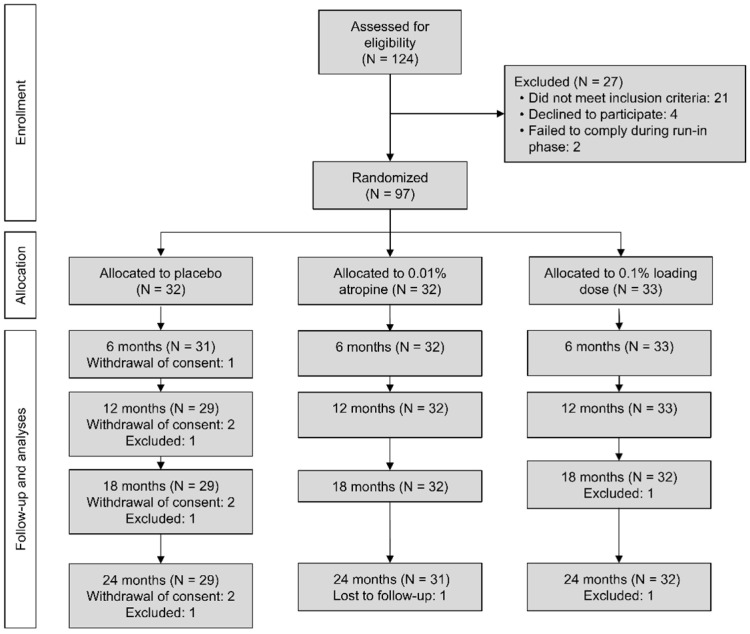
Flow-chart diagram (Consolidated Standards of Reporting Trials, CONSORT) of the study. Abbreviations: 0.1% loading dose, group that received 0.1% for the first six months followed by 0.01% for the following 18 months; 0.01%, group that received 0.01% for two years; N, number.

**Table 3:** Adverse events for the second year of intervention.

Group	Event	12 mo	18 mo	24 mo
0.1% loading dose	Total events, N/total N (%)	2/33 (6%)	2/32 (6%)	5/32 (16%)
	Eye redness/irritation, N/total N (%)	1/33 (3%)	0/32 (0%)	1/32 (3%)
	Photophobia, N/total N (%)	0/33 (0%)	0/32 (0%)	1/32 (3%)
	Blurred near vision, N/total N (%)	0/33 (0%)	0/32 (0%)	1/32 (3%)
	Blurred distance vision, N/total N (%)	0/33 (0%)	0/32 (0%)	0/32 (0%)
	Other, N/total N (%)	1/33 (3%)	2/32 (6%)	2/32 (6%)
	Dilated pupils, N/total N (%)	0/33 (0%)	0/32 (0%)	0/32 (0%)
0.01%	Total events, N/total N (%)	1/32 (3%)	4/32 (13%)	1/31 (3%)
	Eye redness/irritation, N/total N (%)	0/32 (0%)	2/32 (6%)	0/31 (0%)
	Photophobia, N/total N (%)	0/32 (0%)	1/32 (3%)	0/31 (0%)
	Blurred near vision, N/total N (%)	0/32 (0%)	0/32 (0%)	0/31 (0%)
	Blurred distance vision, N/total N (%)	0/32 (0%)	0/32 (0%)	0/31 (0%)
	Other, N/total N (%)	1/32 (3%)	1/32 (3%)	1/31 (3%)
	Dilated pupils, N/total N (%)	0/32 (0%)	0/32 (0%)	0/31 (0%)
Placebo	Total events, N/total N (%)	2/29 (7%)	2/29 (7%)	3/29 (10%)
	Eye redness/irritation, N/total N (%)	0/29 (0%)	1/29 (3%)	1/29 (3%)
	Photophobia, N/total N (%)	0/29 (0%)	0/29 (0%)	1/29 (3%)
	Blurred near vision, N/total N (%)	1/29 (3%)	0/29 (0%)	0/29 (0%)
	Blurred distance vision, N/total N (%)	0/29 (0%)	0/29 (0%)	0/29 (0%)
	Other, N/total N (%)	1/29 (3%)	1/29 (3%)	1/29 (3%)
	Dilated pupils, N/total N (%)	0/29 (0%)	0/29 (0%)	0/29 (0%)

“Total events” indicate the total number of participants who experienced at least one adverse event. Abbreviations: N, number of participants; mo, month.
