# Supplementary material for: Two-Year Results of 0.01% Atropine Eye Drops and 0.1% Loading Dose for Myopia Progression Reduction in Danish Children: A Placebo-Controlled, Randomized Clinical Trial
Source: J Pers Med. 2024 Feb 2;14(2):175. doi: 10.3390/jpm14020175 (PMC10890135; doi:10.3390/jpm14020175)
Supplement: Supplementary file 1 [file jpm-14-00175-s001.zip › Table S1.pdf]

Supplementary Table S1. All Linear Mixed Model Effect Estimates of Treatment Group on Ocular Parameters.

Group / Time point	Placebo	0.1% loading dose[a]	0.01%[b]
**AL, mm**			
Baseline	24.60 (24.42; 24.78)		
6-mo	24.81 (24.62; 25.00)	−0.13 (−0.16; −0.09)	−0.06 (−0.10; −0.02)
p-value		*<0.001**	*0.003**
12-mo	24.94 (24.75; 25.14)	−0.09 (−0.15; −0.04)	−0.07 (−0.13; −0.01)
p-value		*0.002**	*0.02**
18-mo	25.08 (24.87; 25.28)	−0.09 (−0.17; −0.02)	−0.09 (−0.17; −0.01)
p-value		*0.02**	*0.02**
24-mo	25.17 (24.97; 25.38)	−0.08 (−0.17; 0.01)	−0.10 (−0.19; −0.01)
p-value		*0.08*	*0.02**
**SER, diopters**			
Baseline	−2.99 (−3.26; −2.71)		
6-mo	−3.34 (−3.65; −3.04)	0.39 (0.26; 0.53)	0.16 (0.02; 0.29)
p-value		*<0.001**	*0.03**
12-mo	−3.64 (−3.97; −3.31)	0.24 (0.09; 0.39)	0.19 (0.04; 0.34)
p-value		*0.002**	*0.01**
18-mo	−3.92 (−4.26; −3.57)	0.20 (0.01; 0.39)	0.20 (0.01; 0.40)
p-value		*0.04**	*0.04**
24-mo	−4.17 (−4.52; −3.81)	0.12 (−0.10; 0.33)	0.26 (0.04; 0.48)
p-value		*0.30*	*0.02**
**Distance BCVA, LogMAR**			
Baseline	−0.10 (−0.12; −0.09)		
6-mo	−0.12 (−0.14; −0.10)	−0.01 (−0.03; 0.01)	0.01 (−0.01; 0.03)
adjusted-p		*0.77*	*0.77*
12-mo	−0.11 (−0.13; −0.10)	−0.01 (−0.03; 0.01)	−0.01 (−0.03; 0.02)
adjusted-p		*0.71*	*0.85*
18-mo	−0.13 (−0.15; −0.11)	0.00 (−0.02; 0.02)	0.01 (−0.01; 0.04)
adjusted-p		*0.93*	*0.65*
24-mo	−0.12 (−0.14; −0.10)	0.00 (−0.02; 0.02)	0.00 (−0.02; 0.02)
adjusted-p		*0.87*	*0.97*
**Near BCVA, LogMAR**			
Baseline	−0.07 (−0.09; −0.05)		
6-mo	−0.07 (−0.10; −0.05)	0.02 (0.00; 0.05)	0.01 (−0.01; 0.04)
adjusted-p		*0.47*	*0.71*
12-mo	−0.09 (−0.12; −0.07)	0.01 (−0.01; 0.04)	0.03 (0.00; 0.05)
adjusted-p		*0.60*	*0.45*
18-mo	−0.09 (−0.11; −0.06)	0.00 (−0.02; 0.03)	0.01 (−0.02; 0.04)
adjusted-p		*0.84*	*0.77*
24-mo	−0.08 (−0.11; −0.06)	0.00 (−0.02; 0.03)	0.01 (−0.02; 0.04)
adjusted-p		*0.82*	*0.84*
**Accommodation amplitude, diopters**			
Baseline	16.4 (15.6; 17.2)		
6-mo	14.4 (13.5; 15.4)	−4.9 (−6.0; −3.9)	−0.5 (−1.6; 0.6)
adjusted-p		*<0.001**	*0.71*
12-mo	16.7 (15.7; 17.7)	−0.7 (−1.8; 0.4)	−1.1 (−2.2; 0.0)
adjusted-p		*0.57*	*0.43*

18-mo	16.7 (15.8; 17.7)	−0.4 (−1.5; 0.6)	0.2 (−0.9; 1.3)
adjusted-p		0.77	*0.87*
24-mo	16.8 (15.8; 17.8)	−0.5 (−1.5; 0.6)	−0.8 (−1.8; 0.3)
adjusted-p		*0.76*	*0.57*
**IOP, mmHg**			
Baseline	15.8 (15.1; 16.5)		
6-mo	16.8 (15.9; 17.7)	0.2 (−0.8; 1.3)	−0.5 (−1.6; 0.5)
adjusted-p		*0.86*	*0.71*
12-mo	17.3 (16.3; 18.2)	−0.1 (−1.2; 1.0)	0.2 (−0.9; 1.3)
adjusted-p		*0.93*	*0.87*
18-mo	17.7 (16.9; 18.6)	0.3 (−0.7; 1.3)	0.09 (−0.9; 1.1)
adjusted-p		*0.81*	*0.93*
24-mo	17.7 (16.7; 18.6)	0.2 (−0.9; 1.3)	−0.7 (−1.8; 0.5)
adjusted-p		*0.87*	*0.67*
**Mesopic pupil diameter, mm**			
Baseline	4.28 (4.08; 4.49)		
6-mo	4.31 (4.00; 4.62)	1.11 (0.76; 1.47)	0.19 (−0.16; 0.55)
adjusted-p		_<0.001*_	*0.71*
12-mo	4.31 (4.03; 4.59)	0.23 (−0.11; 0.56)	0.11 (−0.23; 0.44)
adjusted-p		*0.57*	*0.80*
18-mo	4.33 (4.07; 4.60)	0.12 (−0.18; 0.42)	−0.03 (−0.33; 0.27)
adjusted-p		*0.77*	*0.92*
24-mo	4.35 (4.10; 4.60)	−0.04 (−0.32; 0.23)	0.09 (−0.18; 0.37)
adjusted-p		*0.87*	*0.80*
**Photopic pupil diameter, mm**			
Baseline	2.80 (2.67; 2.94)		
6-mo	2.70 (2.47; 2.92)	1.04 (0.77; 1.31)	0.13 (−0.14; 0.40)
adjusted-p		_<0.001*_	*0.71*
12-mo	2.76 (2.58; 2.94)	0.17 (−0.05; 0.38)	0.07 (−0.14; 0.28)
adjusted-p		*0.53*	0.80
18-mo	2.74 (2.49; 2.99)	0.06 (−0.14; 0.26)	−0.06 (−0.26; 0.13)
adjusted-p		*0.83*	*0.80*
24-mo	2.77 (2.62; 2.91)	0.03 (−0.14; 0.19)	0.06 (−0.10; 0.23)
adjusted-p		*0.87*	*0.77*
**Sub-foveal choroidal thickness, μm**			
Baseline	242 (228; 257)		
6-mo	239 (224; 255)	13 (6; 21)	2 (−6; 10)
adjusted-p		_0.01*_	*0.85*
12-mo	245 (229; 261)	2 (−5; 9)	5 (−2; 13)
adjusted-p		0.81	0.56
18-mo	241 (225; 257)	2 (−6; 11)	4 (−5; 13)
adjusted-p		*0.85*	*0.71*
24-mo	242 (225; 259)	5 (−4; 14)	7 (−2; 16)
adjusted-p		*0.68*	*0.49*
**ACD, mm**			
Baseline	3.30 (3.25; 3.35)		
6-mo	3.31 (3.26; 3.37)	*0.05 (0.03; 0.06)*	0.02 (0.00; 0.03)
adjusted-p		_<0.001*_	*0.43*
12-mo	3.31 (3.26; 3.37)	*0.03 (0.02; 0.05)*	0.01 (0.00; 0.03)
adjusted-p		_0.001*_	*0.45*

18-mo	3.33 (3.28; 3.38)	0.02 (0.01; 0.04)	0.01 (0.00; 0.03)
adjusted-p		*0.10*	*0.57*
24-mo	3.34 (3.29; 3.39)	0.02 (0.00; 0.03)	0.02 (0.00; 0.03)
adjusted-p		*0.40*	*0.40*
**Iridocorneal angle, degrees**			
Baseline	43.9 (42.7; 45.2)		
6-mo	45.2 (43.5; 47.1)	−4.0 (−5.7; −2.2)	−1.2 (−3.0; 0.6)
adjusted-p		*<0.001**	*0.57*
12-mo	45.0 (43.5; 46.5)	−1.1 (−2.6; 0.3)	−1.1 (−2.5; 0.4)
adjusted-p		*0.53*	*0.56*
18-mo	44.6 (43.0; 46.2)	−0.9 (−2.3; 0.4)	−0.6 (−2.0; 0.7)
adjusted-p		*0.57*	*0.71*
24-mo	44.9 (43.3; 46.4)	−0.5 (−1.9; 0.9)	−0.7 (−2.1; 0.7)
adjusted-p		*0.80*	*0.71*
**CCT, μm**			
Baseline	551 (545; 558)		
6-mo	554 (547; 560)	0 (−2; 2)	−2 (−4; 1)
adjusted-p		*0.97*	*0.43*
12-mo	554 (548; 561)	0 (−2; 2)	−2 (−4; 1)
adjusted-p		*1.00*	*0.49*
18-mo	553 (546; 560)	−1 (−3; 1)	1 (−2; 3)
adjusted-p		*0.79*	*0.85*
24-mo	553 (547; 560)	0 (−2; 2)	1 (−2; 4)
adjusted-p		*0.90*	*0.77*
**K1 (front), diopters**			
Baseline	43.03 (42.72; 43.34)		
6-mo	42.99 (42.67; 43.30)	−0.01 (−0.05; 0.03)	0.01 (−0.03; 0.05)
adjusted-p		*0.86*	*0.85*
12-mo	42.91 (42.60; 43.22)	0.00 (−0.04; 0.04)	0.02 (−0.03; 0.06)
adjusted-p		*1.00*	*0.79*
18-mo	42.86 (42.55; 43.18)	0.03 (−0.03; 0.09)	0.03 (−0.05; 0.09)
adjusted-p		*0.71*	*0.71*
24-mo	42.88 (42.57; 43.20)	0.04 (−0.03; 0.10)	0.00 (−0.06; 0.07)
adjusted-p		*0.71*	*0.94*
**K2 (front), diopters**			
Baseline	43.89 (43.57; 44.21)		
6-mo	43.88 (43.56; 44.21)	0.03 (−0.03; 0.09)	0.02 (−0.05; 0.08)
adjusted-p		0.71	*0.85*
12-mo	43.85 (43.52; 44.20)	0.05 (−0.01; 0.11)	0.02 (−0.03; 0.08)
adjusted-p		*0.45*	*0.77*
18-mo	43.84 (43.51; 44.17)	0.05 (−0.02; 0.11)	0.01 (−0.06; 0.08)
adjusted-p		*0.57*	*0.87*
24-mo	43.88 (43.55; 44.21)	0.06 (−0.02; 0.13)	0.00 (−0.08; 0.07)
adjusted-p		*0.56*	*0.94*
**Lens thickness, mm**			
Baseline	3.32 (3.28; 3.35)		
6-mo	3.32 (3.29; 3.36)	−0.02 (−0.04; 0.00)	−0.01 (−0.03; 0.01)
adjusted-p		*0.16*	*0.57*
12-mo	3.32 (3.28; 3.36)	0.00 (−0.04; 0.04)	0.00 (−0.03; 0.01)
adjusted-p		*1.00*	*0.87*

18-mo	3.32 (3.29; 3.36)	0.00 (−0.01; 0.02)	0.00 (−0.02; 0.01)
adjusted-p		0.87	*0.83*
24-mo	3.33 (3.29; 3.36)	−0.01 (−0.03; 0.01)	−0.02 (−0.04; 0.00)
adjusted-p		*0.63*	*0.43*

Effect estimates for the placebo group are total to the given time point while effect estimates for the intervention groups (0.1% loading dose and 0.01%) are differences from the placebo group at the given time point. Significance levels for exploratory secondary outcomes were reported as adj-p while primary outcomes determining treatment efficacy were excluded from FDR-adjustment and reported as p. Abbreviations: ACD, anterior chamber depth; AL, axial length; BCVA, best-corrected visual acuity; CCT, central corneal thickness; IOP, intra-ocular pressure; K1, the flat corneal meridian; K2, the steep corneal meridian; mo, months; p, p-value; adjusted-p, p-value adjusted by False Discovery Rate; SER, Spherical equivalent refraction.

[a] Change in the 0.1% loading dose group compared to placebo at the given time point.

[b] Change in the 0.01% group compared to placebo at the given time point.

* Statistically significant if below our p-value or adjusted-p cut-off of 0.05.
