# Supplementary material for: Two-Year Results of 0.01% Atropine Eye Drops and 0.1% Loading Dose for Myopia Progression Reduction in Danish Children: A Placebo-Controlled, Randomized Clinical Trial
Source: J Pers Med. 2024 Feb 2;14(2):175. doi: 10.3390/jpm14020175 (PMC10890135; doi:10.3390/jpm14020175)
Supplement: Supplementary file 1 [file jpm-14-00175-s001.zip › Table S2.pdf]

Supplementary Table S2. Adverse Events.

Group	Event	3mo	6mo	9mo	12mo	18mo	24mo
**0.1% loading dose**	Total events, N/total N (%)	22/33 (66%)	15/33 (45%)	6/33 (18%)	2/33 (6%)	2/32 (6%)	5/32 (16%)
	Eye redness/irritation, N/total N (%)	3/33 (9%)	2/33 (6%)	0/33 (0%)	1/33 (3%)	0/32 (0%)	1/32 (3%)
	Photophobia, N/total N (%)	16/33 (48%)	11/33 (33%)	2/33 (6%)	0/33 (0%)	0/32 (0%)	1/32 (3%)
	Blurred near vision, N/total N (%)	18/33 (55%)	12/33 (36%)	1/33 (3%)	1/33 (3%)	0/32 (0%)	1/32 (3%)
	Blurred distance vision, N/total N (%)	0/33 (0%)	0/33 (0%)	0/33 (0%)	0/33 (0%)	0/32 (0%)	0/32 (0%)
	Other, N/total N (%)	3/33 (9%)	2/33 (6%)	2/33 (6%)	0/33 (0%)	2/32 (6%)	2/32 (6%)
	Dilated pupils, N/total N (%)	11/33 (33%)	7/33 (21%)	1/33 (3%)	0/33 (0%)	0/32 (0%)	0/32 (0%)
**0.01%**	Total events, N/total N (%)	8/32 (25%)	1/32 (3%)	1/32 (3%)	1/32 (3%)	4/32 (13%)	1/32 (3%)
	Eye redness/irritation, N/total N (%)	2/32 (6%)	1/32 (3%)	1/32 (3%)	0/32 (0%)	2/32 (6%)	0/32 (0%)
	Photophobia, N/total N (%)	3/32 (9%)	0/32 (0%)	0/32 (0%)	0/32 (0%)	1/32 (3%)	0/32 (0%)
	Blurred near vision, N/total N (%)	0/32 (0%)	1/32 (3%)	0/32 (0%)	1/32 (3%)	0/32 (0%)	0/32 (0%)
	Blurred distance vision, N/total N (%)	0/32 (0%)	0/32 (0%)	0/32 (0%)	0/32 (0%)	0/32 (0%)	0/32 (0%)
	Other, N/total N (%)	5/32 (16%)	0/32 (0%)	0/32 (0%)	1/32 (3%)	1/32 (3%)	1/32 (3%)
	Dilated pupils, N/total N (%)	0/32 (0%)	0/32 (0%)	0/32 (0%)	0/32 (0%)	0/32 (0%)	0/32 (0%)
**Placebo**	Total events, N/total N (%)	6/32 (19%)	4/31 (13%)	1/30 (3%)	1/29 (3%)	2/29 (7%)	3/28 (11%)
	Eye redness/irritation, N/total N (%)	2/32 (6%)	2/31 (6%)	1/30 (3%)	0/29 (0%)	1/29 (3%)	1/28 (4%)
	Photophobia, N/total N (%)	0/32 (0%)	0/31 (0%)	0/30 (0%)	0/29 (0%)	0/29 (0%)	1/28 (4%)
	Blurred near vision, N/total N (%)	0/32 (0%)	0/31 (0%)	0/30 (0%)	1/29 (3%)	0/29 (0%)	0/28 (0%)
	Blurred distance vision, N/total N (%)	0/32 (0%)	0/31 (0%)	0/30 (0%)	0/29 (0%)	0/29 (0%)	0/28 (0%)

	Other, N/total N (%)	4/32 (13%)	3/31 (10%)	0/30 (0%)	1/29 (3%)	1/29 (3%)	1/28 (4%)
	Dilated pupils, N/total N (%)	0/32 (0%)	0/31 (0%)	0/30 (0%)	0/29 (0%)	0/29 (0%)	0/28 (0%)

"Total events" refers to the number of participants with one or more adverse events. Abbreviations: N, Number; mo, month.
